# Inhibiting C-Reactive Protein for the Treatment of Cardiovascular Disease: Promising Evidence from Rodent Models

**DOI:** 10.1155/2014/353614

**Published:** 2014-04-02

**Authors:** Alexander J. Szalai, Mark A. McCrory, Dongqi Xing, Fadi G. Hage, Andrew Miller, Suzanne Oparil, Yiu-Fai Chen, Michelle Mazzone, Richard Early, Scott P. Henry, Thomas A. Zanardi, Mark J. Graham, Rosanne M. Crooke

**Affiliations:** ^1^Division of Clinical Immunology and Rheumatology, Department of Medicine, The University of Alabama at Birmingham, 1825 University Boulevard, SHEL 214, Birmingham, Al 35294-2182, USA; ^2^Division of Cardiovascular Disease, The University of Alabama at Birmingham, Birmingham, AL 35294-0006, USA; ^3^Charles River Laboratories, Sparks, NV 89431, USA; ^4^Isis Pharmaceuticals, 2855 Gazelle Court, Carlsbad, CA 92008, USA

## Abstract

Raised blood C-reactive protein (CRP) level is a predictor of cardiovascular events, but whether blood CRP is causal in the disease process is unknown. The latter would best be defined by pharmacological inhibition of the protein in the context of a randomized case-control study. However, no CRP specific drug is currently available so such a prospective study cannot be performed. Blood CRP is synthesized primarily in the liver and the liver is an organ where antisense oligonucleotide (ASO) drugs accumulate. Taking advantage of this we evaluated the efficacy of CRP specific ASOs in rodents with experimentally induced cardiovascular damage. Treating rats for 4 weeks with a rat CRP-specific ASO achieved >60% reduction of blood CRP. Notably, this effect was associated with improved heart function and pathology following myocardial infarction (induced by ligation of the left anterior descending artery). Likewise in human CRP transgenic mice treated for 2 weeks with a human CRP-specific ASO, blood human CRP was reduced by >70% and carotid artery patency was improved (2 weeks after surgical ligation). CRP specific ASOs might pave the way towards a placebo-controlled trial that could clarify the role of CRP in cardiovascular disease.

## 1. Introduction

C-reactive protein (CRP), the prototypic acute phase reactant, is produced primarily by the liver as part of the body's mechanism to restrict injury and promote repair after an inflammation evoking injury [1–3]. CRP is a member of the phylogenetically ancient and evolutionarily conserved pentraxin family of proteins and consists of five noncovalently bound subunits, each of 206 amino acids, arranged symmetrically around a central pore [4]. The molecule has a ligand recognition face that contains a Ca^2+^-dependent binding site, and an effector molecule binding face that is capable of initiating fluid phase pathways of host defence (by activating the complement system) and cell-mediated ones (by activating complement or binding to Fc receptors) [4]. Regulation of CRP expression occurs mostly at the transcriptional level, with interleukin 6 (IL-6) being its major inducer and interleukin 1 (IL-1) synergistically enhancing the IL-6 effect [4, 5]. The rise in blood CRP after tissue injury is rapid, with levels increasing by as much as 1000-fold above baseline within 24 hours. This plasticity makes blood CRP an ideal clinical marker of a patient's general health status, a purpose for which it has been used for half a century [1–6].

Since the early 1980s, largely because of increasingly widespread use of automated high sensitivity CRP assays, clinicians and physician scientists have been able to reproducibly and accurately measure the low levels of blood CRP (≤3 mg/L) routinely seen in ostensibly healthy people. This capacity has led to accumulation of extensive observational data linking CRP to various kinds of disease [6–10]. The relationship of CRP to the inflammatory aspects of cardiovascular disease (CVD) has been an area of keen interest. Indeed, based upon multiple prospective epidemiological studies, CRP is now recognized as an independent marker and powerful predictor for future risks of myocardial infarction (MI), stroke, and death from coronary heart disease (CHD) in individuals apparently free of known CVD. Further, data from at least four clinical trials (PROVE IT-TIMI, REVERSAL, JUPITER, and SATURN) suggest a role for CRP in the atherogenic process [11–14]. In these studies, the indirect reduction of blood CRP levels that accompanied treatment with statins was found to be independently and significantly related to event-free survival and/or decreased progression of documented coronary disease and/or major cardiovascular events. In patients where low density lipoprotein cholesterol (LDL-C) alone was reduced, disease progression was slowed by statin therapy, but in patients where LDL-C and CRP were both reduced, atheroma progression was halted. Furthermore in at-risk patients given maximally intensive statin therapy, lowering of CRP was associated with atheroma regression [14].

Based on the known biology of CRP it would not be a surprise if the protein was ultimately found to contribute to the pathophysiological processes leading to CVD. For example various studies demonstrate that CRP can activate complement and endothelial cells and promote their dysfunction [15–17]. Others show that CRP is detected in early atherosclerotic lesions [17, 18] and that it is colocalized with activated complement components and enzymatically degraded LDL in human vascular lesions isolated by atherectomy [19–21]. In addition there is compelling direct evidence from multiple transgenic models indicating that human CRP has a pathogenic role in vascular disease [22–25]. Despite these data, generated independently by many different groups, the exact biological role of CRP in CVD in humans and the overall importance of its contribution therein remains equivocal [26] because there is no way to selectively reduce CRP in patients. Towards solving this nagging problem a small molecule inhibitor of human CRP, 1,6-bis(phosphocholine)-hexane, was synthesized and tested in a preclinical rodent model* in vivo *[27]. The compound is designed to crosslink two CRP molecules, thereby increasing the protein's clearance and blocking its ability to bind endogenous ligands. It was shown that in rats that were administered human CRP by injection, administration of this compound ameliorated human CRP associated exacerbation of MI caused by ligation of the coronary artery [27]. Despite the fact this was a xenogenic system, the study demonstrated for the first time that therapeutic inhibition of CRP might be a promising new approach for cardioprotection in acute MI. Still, to definitively address the question of causality of CRP in the pathogenesis of CVD and whether reduction of CRP would result in a meaningful decrease in adverse CVD outcomes, the use of a specific pharmacological inhibitor of endogenously expressed CRP is preferred.

Antisense oligonucleotides (ASOs) are highly specific agents that can be used therapeutically to prevent the translation of disease-associated proteins, an effect achieved via selective degradation of targeted mRNAs [28]. Because of their specificity and propensity to distribute to the liver, where CRP is synthesized and secreted [3–5], ASOs provide an efficient means of reducing CRP expression. Using CRP transgenic mice (CRPtg) [29, 30] we previously established that human CRP targeting ASOs are effective for reduction of human CRP and efficacious against collagen-induced arthritis [31]. Herein we employed additional species-specific CRP targeting ASOs and tested their efficacy in two different animal models of CVD wherein a role for CRP has already been established, namely, rats subjected to experimentally induced MI [27] and CRPtg mice subjected to carotid artery ligation [24, 25]. We provide new evidence that ASO-mediated reduction of endogenously expressed rat CRP (in rats) and human CRP (in CRPtg) is efficacious in both diseases. CRP specific ASOs have the potential to be therapeutically beneficial in humans at risk for CVD.

## 2. Materials and Methods

### 2.1. Animals

Eleven-week-old Sprague-Dawley rats were obtained from Charles River Breeding Laboratories, fed a standard rat pellet diet* ad libitum*, and acclimated to local conditions for 1 week before use in any experiments. Wild-type mice (C57BL/6 strain) and littermate CRPtg were from our own colonies. Details of the human CRP transgene and its human-like expression in CRPtg have been described elsewhere [29, 30]. In CRPtg human CRP is present in the blood at concentrations relevant to human disease, that is, low levels under steady-state conditions (<3 *μ*g/mL) and high levels during an acute phase response (>500 *μ*g/mL). All mice were fed a standard mouse pellet diet* ad libitum* and they were 8–12 weeks old when used in experiments. Only male rats and male mice were subjected to experimentation and all were maintained at constant humidity (60 ± 5%) and temperature (24 ± 1°C) with a 12 hour light cycle (6 AM to 6 PM). All protocols were approved by the Institutional Animal Care and Use Committee (IACUC) at the University of Alabama at Birmingham and were consistent with the* Guide for the Care and Use of Laboratory Animals* published by the National Institutes of Health “Public Health Service Policy on Humane Care and Use of Animals, DHEW Publication number 96-01, PHS Policy revised in 2002.”

### 2.2. Antisense Oligonucleotides

ASOs designed to specifically hybridize to either rat or human CRP mRNA were synthesized and purified as described previously [28, 31, 32]. Each ASO was 20 nucleotides in length and comprised a central unmodified core consisting of 10 or 14 nucleotides, flanked by phosphorothioate linkages and three or five 2′-O-methoxyethyl (2′-MOE) modifications on the 3′ and 5′ flanking ends. The ASOs thus had a “3-14-3” or a “5-10-5” configuration. Candidate ASOs were evaluated for their ability to reduce IL-6 stimulated CRP mRNA expression in cultures of rat or human hepatoma cells (data not shown). In these experiments several ASOs significantly reduced CRP mRNA levels, with IC_50_ values for the lead compounds in the 5 nM range (data not shown). Based on their potency in these assays, a rat CRP specific ASO (ISIS 197178) and a human CRP specific ASO (ISIS 353512) were chosen for evaluation* in vivo*. A third ASO (ISIS 141923), which is a scrambled ASO not complementary to any known rat or human gene sequence, served as a control.

### 2.3. Rat Myocardial Infarction Model

After acclimatization, rats were randomly assigned to 3 treatment groups and received for 4 wks the rat CRP-specific ASO ISIS 197178 or the control ASO ISIS 141923 (both at 150 mg/kg/wk i.p.) or vehicle (0.9% NaCl). At the beginning and end of the 4-week treatment phase blood was collected to assess CRP, IL-6, and alanine transaminase (ALT) levels. The next day echocardiography was performed and then the rats were subjected to left anterior descending coronary artery (LADCA) ligation as described [33]. Briefly, rats were anesthetized with ketamine-xylazine (80–15 mg/kg i.p.), intubated, ventilated with a rodent respirator, and laid on a heating pad warmed to 37°C. The heart was exposed via a left intercostal thoracotomy through the 4th intercostal space, and the pericardium removed for identification of the LADCA. The LADCA was ligated 2 mm below the left atrium using a tapered needle and a 5-0 polypropylene ligature. Occlusion was confirmed by a sudden pallor of the anterior wall of the left ventricle (LV). The chest cavity was closed and the rats allowed to recover, with analgesic (0.05 mg/kg buprenorphine s.c.) given twice daily over the next 3 days. One week after LADCA echocardiography was repeated and the rats euthanized with an overdose of ketamine-xylazine. Hearts were then removed, weighed, and cut into 2 mm slices (average of 5 transverse slices/heart at the level below the LADCA) perpendicular to the apex-base axis [33]. Tissue slices were fixed with 4% paraformaldehyde, embedded in paraffin, cut into 5 *μ*m sections, and stained with picrosirius red (0.1%) for assessment of collagen area (an index of replacement fibrosis and infarct size) [33]. Morphometric analysis of tissue sections was carried out by light microscopy with a Qimaging QiCam digital camera (Qimaging) interfaced with a computer system running Metamorph 6.2v4 software (Universal Imaging). For infarct size estimation, the ratio of the picrosirius red-stained area (or the perimeter of that area) divided by the total area of the ventricle (or the perimeter of the ventricle) was calculated for six 5 *μ*m sections taken from 0–2, 2–4, 4–6, 6–8, and 8–10 mm distal from the heart apex.

### 2.4. Echocardiography

Echocardiography was performed on isoflurane anesthetized rats using a Philips Sonos 5500 ultrasound system equipped with a 15 MHz transducer as described previously [33]. After recording heart rate (HR), LV end-systolic dimension (LVESD) and LV end-diastolic dimension (LVEDD) were measured by two-dimensional-guided M-mode imaging from the parasternal short-axis view below the mitral valve. LV end-diastolic volume (EDV), end-systolic volume (ESV), ejection fraction (EF), and fractional shortening (FS) were calculated as follows: EDV = 7 × LVEDD^3^/(2.4 + LVEDD), ESV = 7 × LVESD^3^/(2.4 + LVESD), FS = (LVEDD − LVESD)/LVEDD × 100, and EF = (EDV − ESV)/EDV × 100. The mean velocity of circumferential shortening (VCFR) was also calculated. A single examiner blinded to treatment performed and interpreted all studies.

### 2.5. Mouse Vascular Injury Model

Wild-type versus CRPtg mice were randomly assigned to treatment groups to receive for 2 wks the human CRP-specific ASO 353512 (10, 25, or 50 mg/kg i.p.), the control ASO ISIS 141923 (20 mg/kg/wk i.p.), or an equal volume vehicle (0.9% NaCl). At the beginning and end of the 2 wk treatment phase blood was collected to assess human CRP and IL-6. Following the run-in phase the right common carotid artery (RCCA) was ligated to stimulate neointima generation as described previously [24, 25]. Briefly, the RCCA was exposed through a midline cervical incision and ligated with an 8-0 silk suture just proximal to the bifurcation. The left common carotid artery was surgically exposed but not ligated and served as an internal control. Treatments continued postsurgically for 2 more weeks and then the mice were anesthetized and euthanized with an overdose of pentobarbital. The vasculature was immediately flushed with 0.01 M sodium phosphate buffer (pH 7.4) and perfused with 10% formalin and both carotid arteries were excised, fixed in 10% formalin, embedded in paraffin, and sectioned. Representative serial sections were stained with hematoxylin and eosin (H&E) and examined under a light microscope to locate the ligature site; then additional sections of the artery taken 200, 350, 500, and 700 µm proximal to the ligation site were identified and treated with Verhoeff's elastin stain to enhance the elastic laminae. Sections of the unligated contralateral vessels were obtained and processed in the same fashion (data not shown). Computer-assisted morphometric analysis of digitized images captured from each arterial section was performed with image analysis software (Scion Image). To calculate vessel patency, the cross-sectional area of the vessel lumen was divided by the cross-sectional area of the area bounded by the internal elastic lamina. All measurements were performed by a single examiner blinded to the genotype and treatment of the mice.

### 2.6. Measurement of Serum CRP, IL-6, and ALT

Rat CRP was measured using a commercially available enzyme-linked immunoassay kit and the manufacturer's instructions (EMD Millipore, Darmstadt, Germany) and human CRP was measured by an ELISA as described previously [24, 25]. ALT was determined using INFINITY ALT reagents (Sigma-Aldrich Corp., St. Louis, MO).

### 2.7. Statistical Analysis

Results are expressed as the mean ± SEM without transformation. Pairwise comparisons were done using Student's* t*-tests, and comparisons among multiple experimental groups were performed with one-way ANOVA followed by pairwise multiple comparisons using the protected least-squares difference test. Differences were considered significant when the associated* P* value was <0.05. All statistical analyses were performed using the SigmaStat software package (SigmaStat, Jandel Scientific).

## 3. Results

### 3.1. ASO-Mediated Lowering of Rat CRP Is Beneficial in a Rat Model of Acute MI

**Figure 1:**
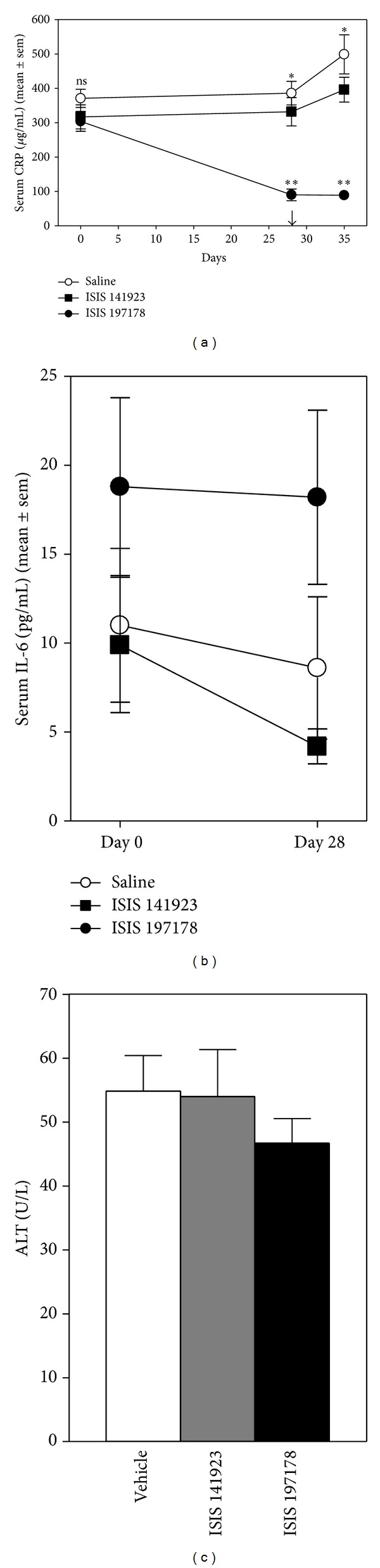
ASO-mediated lowering of CRP in rats subjected to LADCA ligation. (a) Serum CRP values determined by ELISA for rats injected i.p. with a CRP targeting ASO (ISIS 197178; 150 mg/kg/wk, *n* = 19) (■), a control ASO (ISIS 141923; 150 mg/kg/wk, *n* = 9) (**□**), or an equivalent volume of 0.9% saline (vehicle, *n* = 8 rats) (○). The arrow indicates the day LADCA ligation surgery was performed. The single asterisks and “ns” above the curves indicate *P* < 0.0001 or not significant (*P* > 0.05), respectively, for ANOVAs comparing the three treatment groups on each day. The double asterisks below the lines indicate *P* < 0.0001 for protected least-squares difference tests comparing the ISIS 197178 treated group to both other groups. (b) Serum IL-6 determined by ELISA for the rats shown in (a). (c) Serum alanine transaminase (ALT) levels determined by ELISA on day 35.

Blood CRP level in rats (*n* = 36) was 321.9 ± 18.9 *μ*g/mL at baseline, with no significant difference among the three randomly assigned treatment groups (ANOVA). Administration of the rat CRP specific drug ISIS 197178 (*n* = 19 rats) reduced blood CRP by an average 65% - from 303.8 ± 29.5 *μ*g/mL at baseline to 90.4 ± 17.2 *μ*g/mL on day 28 (Figure 1(a)). In comparison, rats treated with saline (*n* = 8) or the control ASO ISIS 141923 (*n* = 9) showed no significant deviation of blood CRP from baseline levels on day 28 (Figure 1(a)). Indeed during this time period CRP levels increased, rather than decreased, in rats receiving saline (10% increase from baseline) and ISIS 141923 (24% increase). IL-6 level at baseline and on day 28 were highly variable with no significant differences among the three treatment groups (ANOVAs) (Figure 1(b)). Importantly, there was no reduction of IL-6 (the major inducer of rat CRP) [34] in rats treated with ISIS 197178 (Figure 1(b)). Both the control and the CRP-specific ASOs were well tolerated as judged by absence of elevation of blood ALT (Figure 1(c)). LADCA ligation on day 28 caused blood CRP to increase by 33% by day 35 for saline-treated rats and by 13% for ISIS 141923-treated rats, but the elevation of CRP was completely blocked for rats receiving ISIS 197178 (Figure 1(a)).

**Figure 2:**
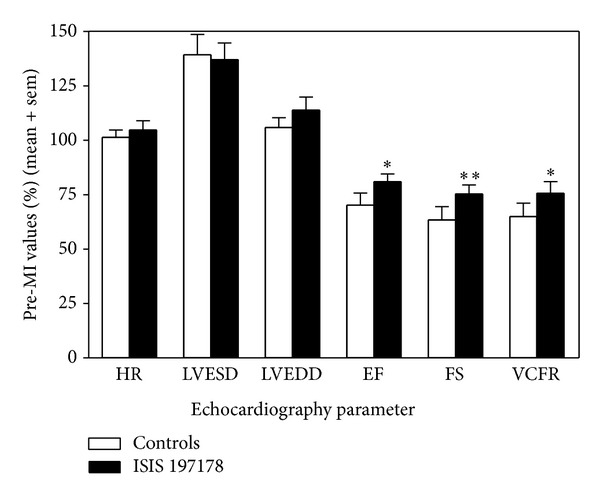
ASO-mediated lowering of CRP in rats subjected to coronary artery ligation improves myocardial infarction (MI) induced cardiac dysfunction. LV function was measured by echocardiographic analysis in rats at baseline and at 1 wk after MI. Heart rate (HR), LV end-systolic dimension (LVESD), LV end-diastolic dimension (LVEDD), ejection fraction (EF), fractional shortening (FS), and the mean velocity of circumferential shortening (VCFR) were calculated as described in the* Materials and Methods*. The results shown are for *n* = 12 rats treated with ISIS 197178 versus *n* = 9 controls (see the* Results* section for details). Single and double asterisks indicate *P* < 0.05 and *P* < 0.005 for unpaired *t*-tests.

**Figure 3:**
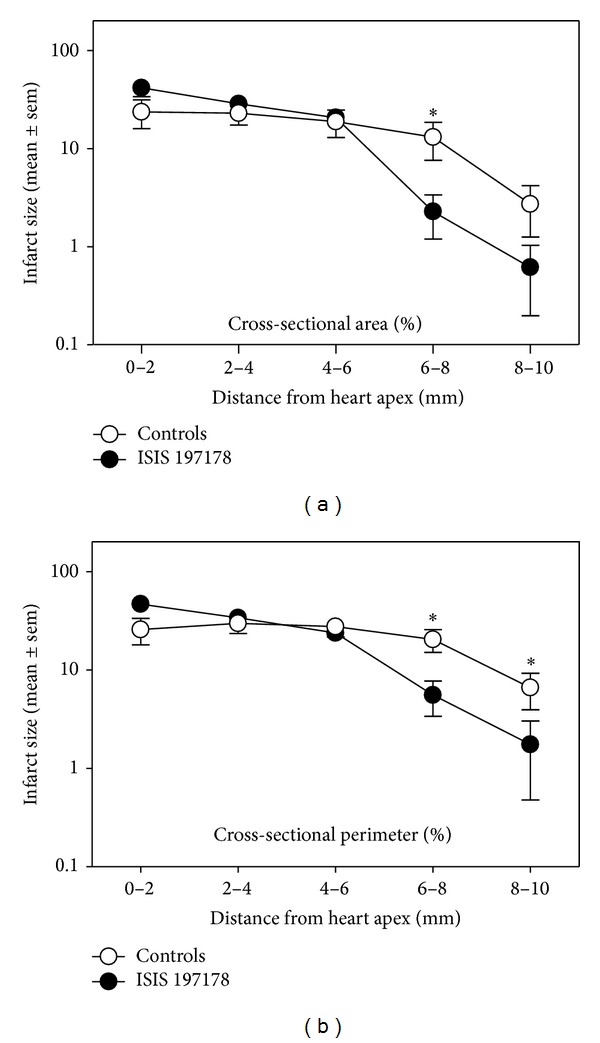
ASO-mediated lowering of CRP in rats subjected to coronary artery ligation reduces infarct size. For hearts from the animals shown in Figure 2, percent infarcted region (area (a) or perimeter (b)) was calculated from picrosirius red-stained LV sections (see Materials and Methods). Rats were injected i.p. with either 0.9% saline or ASO 141923 at 150 mg/kg/wk) (controls, *n* = 9), or an equivalent dose of the CRP targeting ASO ISIS 197178 (*n* = 12). The asterisks indicate *P < 0.05 for unpaired* t*-tests.

After eliminating rats that died during surgery or within hours after LADCA ligation due to lethal MI (3/19 ISIS 197178 treated rats, 3/9 ISIS 141923 treated rats, and 2/8 saline treated rats) and after inspecting post-MI echocardiograms and stained heart sections to eliminate rats in which the LADCA was not properly ligated so no MI was achieved (4/16 ISIS 197178 treated rats, 1/6 ISIS 141923 treated rats, and 2/6 saline treated rats), 21 rats remained for further study. For statistical purposes the remaining saline-treated and ISIS 141923-treated animals (*n* = 9 in total) were pooled and compared to the remaining ISIS 197178-treated rats (*n* = 12). Echocardiographic indices in this subset were consistent with a subtle but significant improvement in post-MI cardiac function for rats treated with ISIS 197178, which showed a 14–23% improvement in EF, FS, and VCFR (Figure 2). Consistent with the echocardiography findings, infarct size was reduced in rats treated with ISIS 197178 compared to controls (Figure 3), with a small but significant zone of protection observed proximal to the site of LADCA ligation (i.e., 6–10 mm from the apex of the heart) (Figure 3).

These results show that for rats treated with ISIS 197178, an ASO that targets rat CRP mRNA and thereby lowers circulating CRP protein level effectively without causing toxicity or inflammation, cardiac dysfunction resulting from experimentally induced acute MI was reduced.

### 3.2. ASO-Mediated Lowering of Human CRP Is Beneficial in a CRPtg Mouse Model of Acute Vascular Injury

In these experiments CRPtg mice were treated for 2 weeks with different doses of a human CRP specific ASO (ISIS 353512; doses of 10, 25, or 50 mg/kg/week i.p.) versus a control ASO (ISIS 141923 at 20 mg/kg/week i.p.) or vehicle (0.9% saline). Following this 2-week run-in phase the RCCA was ligated to stimulate neointima generation. Therapy continued for 2 more weeks and the ligated vessels were harvested to quantitate the effect of human CRP lowering on the blood vessel injury response.

**Figure 4:**
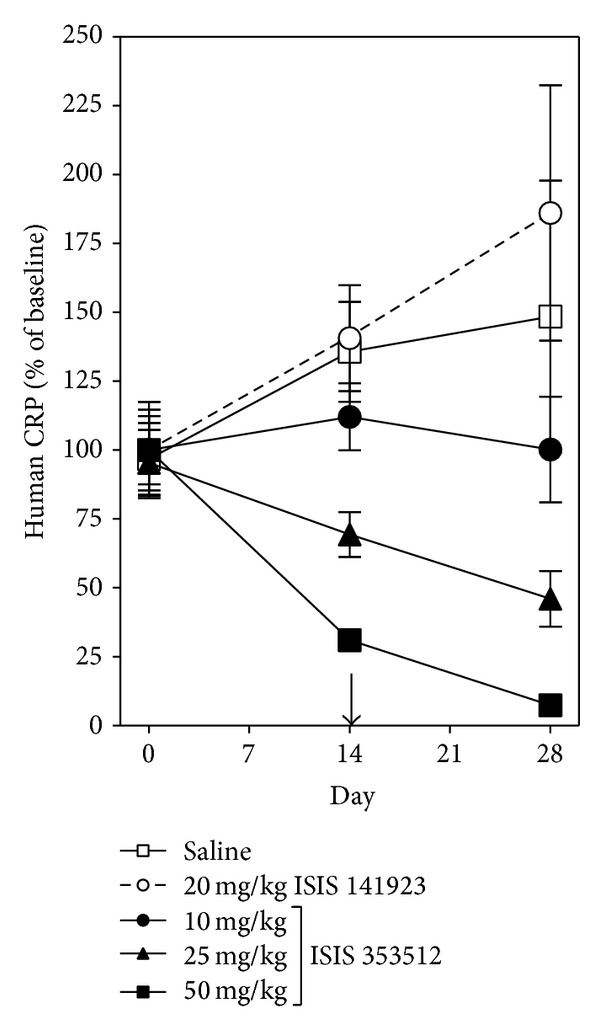
ASO-mediated lowering of human CRP in CRPtg mice subjected to RCCA ligation. Serum human CRP values were determined by ELISA for rats injected i.p. with (i) the CRP targeting ASO ISIS 353512 (10 mg/kg/wk, *n* = 9; 25 mg/kg/wk, *n* = 30; 50 mg/kg/wk, *n* = 5), (ii) the control ASO ISIS 141923 (20 mg/kg/wk, *n* = 12), or (iii) an equivalent volume of 0.9% saline (*n* = 13). The arrow indicates the day RCCA ligation surgery was performed.

Serum human CRP level in CRPtg mice (*n* = 69) was 12.7 ± 1.03 *μ*g/mL at baseline with no statistically significant difference (ANOVA) among the various treatment groups. For both saline-treated and ISIS 141923-treated CRPtg mice, serum human CRP level increased above baseline by ~30% by day 14 and by ~40–85% by day 28 (two weeks after RCCA ligation) (Figure 4). In contrast for mice receiving ISIS 353512, the increase in human CRP level was prevented at the 10 mk/kg/wk dose and reversed in a dose-dependent fashion at the higher doses (Figure 4). Like the ASO we tested in rats, the human CRP specific ASO ISIS 353512 neither reduced serum IL-6 nor raised serum ALT levels in CRPtg mice (data not shown). Furthermore, ISIS 353512 did not reduce mouse CRP levels (data not shown) [31].

**Figure 5:**
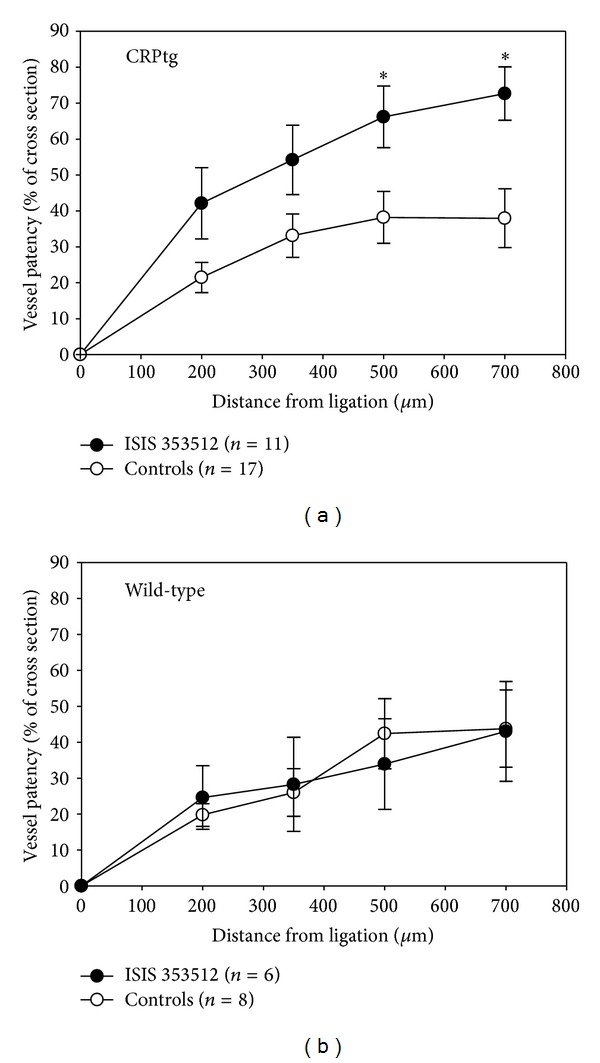
Treatment with the human CRP-specific ASO ISIS 353512 improves blood vessel patency following RCCA ligation in CRPtg mice (a) but not in wild-type mice (b). Blood vessel patency was calculated as described in the* Materials and Methods*. The asterisks indicate *P < 0.05 for unpaired* t*-tests.

For CRPtg mice subjected to surgery, patency of the ligated RCCA was significantly improved by treatment with the human CRP lowering ASO ISIS 353512 (Figure 5(a)). The protective effect was most pronounced distal to the ligature. In stark contrast ISIS 353512 had no effect on ligated RCCA patency in mice that did not express human CRP (Figure 5(b)).

These results show that for CRPtg mice treated with ISIS 353512, an ASO that targets human CRP mRNA and thereby lowers circulating human CRP protein level effectively without causing toxicity or inflammation, carotid artery stenosis resulting from blood vessel ligation is reduced.

## 4. Discussion

The association of elevated baseline CRP to increased risk of CHD is widely recognized [35–37], but it is not known if this association is causal. Furthermore if the association is causal it is not known which of the many biological actions of CRP might support the effect. Notwithstanding the many informative studies of CRP biology performed* in vitro* and in animal models, a clinically approved and specific inhibitor of human CRP will be needed before a true understanding of the physiologic role of CRP in humans at risk of CHD can be ascertained. If treatment of at-risk patients with a CRP lowering drug is found to lower the risk, then that would certainly settle the ongoing debate about whether CRP plays a role in cardiovascular disease. On the other hand even if lowering CRP has no protective effect, the predictive association would still remain.

A small molecule inhibitor of CRP [1,6-bis(phosphocholine)-hexane] that occludes the ligand-binding “B” face of CRP and thereby reportedly blocks its ability to activate complement was tested preclinically [27]. Since (i) complement activation is known to occur in concert with cardiovascular disease [20, 21], (ii) complement activation products are found in vascular lesions associated with cardiovascular disease [19], and (iii) CRP is known to activate complement and colocalize with complement fragments deposited in vascular lesions [19], then administration of 1,6-bis(phosphocholine)-hexane should be of benefit in CVD. Indeed in rats, this compound was shown to prevent the exacerbating effect of exogenously administered human CRP on experimentally induced MI [27]. However, since CRP also interacts with Fc*γ*Rs [4], treatment with 1,6-bis(phosphocholine)-hexane may not completely block all of the potentially detrimental functions of the protein. Also it is not clear what consequences circulating CRP decamers (two pentamers crosslinked by 1,6-bis(phosphocholine)-hexane) might have on the vasculature, since presumably these complexes could be deposited and potentially cause autoimmune or inflammatory side effects. Therefore, despite the fact that this drug improved infarct size and improved cardiac function in rats receiving human CRP, these shortcomings could ultimately limit its therapeutic potential in man.

Our CRP lowering tactic was different. Rather than depleting the circulating protein from the blood, the ASOs we used inhibit CRP production by specifically and selectively preventing the translation of CRP mRNAs. The ASO approach has been successfully used to target proteins not readily amenable to small molecule or antibody based therapeutic interventions. For example Kynamro, an antisense inhibitor of apolipoprotein B [32], the principal apoprotein present on all atherogenic lipids [38], was approved by the FDA in January 2013 for use as an adjunct to first-line lipid-lowering therapies in homozygous familial hypercholesterolemia [39–41]. Because ASOs have much longer half-lives compared to small molecule inhibitors [41, 42], ASOs can be administered more infrequently to patients. Furthermore, CRP is well suited for inhibition using ASO technology because the protein is synthesized primarily by hepatocytes [3, 4, 29, 34], cells that readily accumulate antisense drugs and are sensitive to ASO pharmacology [42–44]. ASOs also accumulate in extrahepatic cells and tissues known to make CRP, such as the kidney, alveolar macrophages, and adipocytes, [19, 37, 42].

The ASOs we tested here were designed to target rat CRP and human CRP (in CRPtg mice). Each ASO was well tolerated at all doses and in both species tested, and both CRP targeting inhibitors specifically reduced their respective CRP serum levels after only short-term administration with a modest dosing regimen. We did not test complement levels in the current study, but we have shown previously that ASO mediated lowering of human CRP is accompanied by reduced complement activation in CRPtg mice [45], an effect like that of 1,6-bis(phosphocholine)-hexane in rats [27]. Notably, ASO-mediated reduction of CRP in both species was not due to reduction of IL-6 and each of the tested ASOs blunted (or reversed) the rise in serum CRP caused by surgery. The proven ability of antisense inhibitors to reduce baseline expression of rat CRP in rats and human CRP in CRPtg mice and to block CRP upregulation after surgery, after treatment for a short duration with only low doses of antisense drugs, suggests that these agents should be useful for intervention in both chronic and acute disease processes. Indeed, we have previously shown that another human CRP specific ASO (ISIS 329993) is efficacious in a CRPtg mouse model of inflammatory arthritis [31]. We have shown here for the first time that, in both rats and mice, ASO-mediated lowering of CRP results in improved outcomes following cardiovascular insult.

Despite a body of evidence that CRP level is a fairly strong predictor of CVD, there is still no agreement on how to integrate CRP measurement into clinical practice or indeed whether it should even routinely be evaluated [46–48]. The main reason is that it is still unknown whether CRP plays a pathophysiologic role in CVD in humans. Without a human specific-CRP drug it has not been possible to conduct clinical trials to test the CRP-CVD hypothesis. The CRP-specific ASO inhibitors that we describe here could fill this gap and provide the impetus for future* in vivo* pharmacological, toxicological, and ultimately clinical studies that will help clearly delineate the role of CRP in CVD in humans. If future studies do confirm a role for CRP in CVD, then perhaps a reasonable ASO treatment group would be patients whose admission values of CRP are 10 mg/L or greater, as these are the patients at highest risk for death and MI [48].
